# Tumor CTR1 and serum copper dynamics reveal a coordinated copper axis linked to high-grade triple-negative breast cancer biology

**DOI:** 10.64898/2025.12.18.25342516

**Published:** 2025-12-27

**Authors:** Vinit C. Shanbhag, Nikita Gudekar, Muhammad Yasir, Kristyn Conrad, Samuel Anakpeba-Dinguyella, Parshad Sutar, Praveen Rao, Michael Petris, Linda Vahdat, Christos Papageorgiou

**Affiliations:** 1 Department of Biochemistry, Life Sciences Center, University of Missouri, Columbia, Missouri, USA.; 2 Ellis Fischel Cancer Center, University of Missouri, Columbia, Missouri, USA.; 3 Department of Electrical Engineering and Computer Science, College of Engineering, University of Missouri, Columbia, Missouri, USA.; 4 Department of Ophthalmology, MU School of Medicine, University of Missouri, Columbia, Missouri, USA.; 5 Dartmouth Cancer Center, Dartmouth Hitchcock Medical Center, Geisel School of Medicine at Dartmouth, Lebanon, New Hampshire, USA.

## Abstract

**Background:**

Copper is an essential nutrient required for energy production, antioxidant defense, and connective tissue maturation, yet has emerged as a metabolic vulnerability in cancer. CTR1 (*SLC31A1*), the high-affinity copper importer, mediates cellular copper uptake, and its upregulation may signal increased copper demand in tumor cells. The dynamics of copper regulation across tumor growth, aggressiveness, and treatment resistance remain poorly defined in breast cancer. We investigated whether CTR1 expression and systemic copper changes reflect a coordinated tumor-systemic copper axis.

**Methods:**

A retrospective dataset of 1632 breast cancer patients receiving neoadjuvant chemotherapy was analyzed to compare CTR1 gene expression between responders and non-responders across molecular subtypes and tumor grades. Findings were extended to a prospective neoadjuvant cohort in which paired pre- and post-treatment serum copper levels were measured. ΔCopper (post–pre change) was correlated with subtype, grade, response, and tumor size.

**Results:**

CTR1 expression was significantly higher in triple-negative breast cancer (TNBC) non-responders than responders (*P* = 0.0021), particularly in grade 3 tumors (*P* = 0.0035), with no difference in luminal subtypes. In the prospective cohort, ΔCopper was positive predominantly in TNBC and strongly grade-dependent: all grade 3 TNBCs exhibited copper elevation post-therapy, whereas all grade 2 TNBCs showed negative ΔCopper (*P* = 0.034). The only relapse in the cohort, a TNBC non-responder, exhibited persistently positive ΔCopper at follow-up and relapse, whereas non-responders from other subtypes showed near-zero or negative ΔCopper (*P* = 0.011). Baseline serum copper was higher in patients with smaller (clinical T1) versus larger (T2–T3) tumors (*P* = 0.033).

**Conclusions:**

Parallel CTR1 upregulation in tumors and systemic copper elevation post-therapy suggest a coordinated copper mobilization program in high-grade TNBC. These integrated retrospective and prospective findings link copper transport to therapy response and tumor aggressiveness, highlighting copper biology as a potential therapeutic axis in breast cancer.

## 1. Background

Breast cancer remains a leading cause of cancer-related morbidity and mortality worldwide, with heterogeneous outcomes across molecular subtypes. Despite advances in targeted therapy and immunotherapy, there is an unmet need for biomarkers that can both capture disease biology in real time and inform treatment decisions.

Copper (Cu) is an essential nutrient required for energy production, antioxidant defense, and connective tissue maturation, yet its metabolism has emerged as a unique vulnerability in cancer.^1^ Cellular copper uptake is primarily mediated by the high-affinity transporter CTR1 (*SLC31A1*), the principal gateway for copper entry and a key regulator of intracellular copper availability.^2, 3^ Global Ctr1 deletion in mice causes embryonic lethality,^4^ and intestinal loss results in systemic copper deficiency,^5^ underscoring its physiological importance. CTR1-driven copper uptake supports several oncogenic programs. Copper-dependent activation of the MAPK–ERK pathway and ULK1/2 signaling links CTR1-mediated copper transport to cell growth, autophagy, and tumor progression.^6, 7^

Elevated serum copper has been associated with adverse prognosis, and intracellular copper availability supports multiple tumor-promoting pathways.^1^ These include angiogenesis through VEGF signaling,^8^ mitochondrial respiration via cytochrome c oxidase,^9, 10^ antioxidant defense through superoxide dismutase,^11, 12^ extracellular matrix remodeling through lysyl oxidases,^13–15^ and regulation of signaling kinases such as MEK1 and ULK1.^6, 7^ In an effort to counteract these oncogenic effects of copper, a phase II clinical study of systemic copper depletion with tetrathiomolybdate (TTM) in high-risk breast cancer patients demonstrated feasibility and suggested benefit in triple-negative breast cancer (TNBC).^16^

Despite these insights, how copper mobilization and CTR1-mediated uptake are regulated across tumor grades, aggressiveness, and treatment contexts remains poorly understood. Most prior studies have examined static baseline copper levels rather than dynamic changes in copper transport or systemic copper availability. To address this gap, we conducted a large retrospective analysis assessing post-chemotherapy CTR1 expression in responders versus non-responders across breast cancer subtypes and tumor grades, complemented by a prospective exploratory study measuring paired pre- and post-treatment serum copper levels (ΔCopper) in the neoadjuvant setting. This integrative approach enables parallel evaluation of tumor copper demand and systemic copper mobilization, providing insight into a coordinated tumor-systemic copper axis in breast cancer.

## 2. Methods

### 2.1 Study design

**Figure 1.**
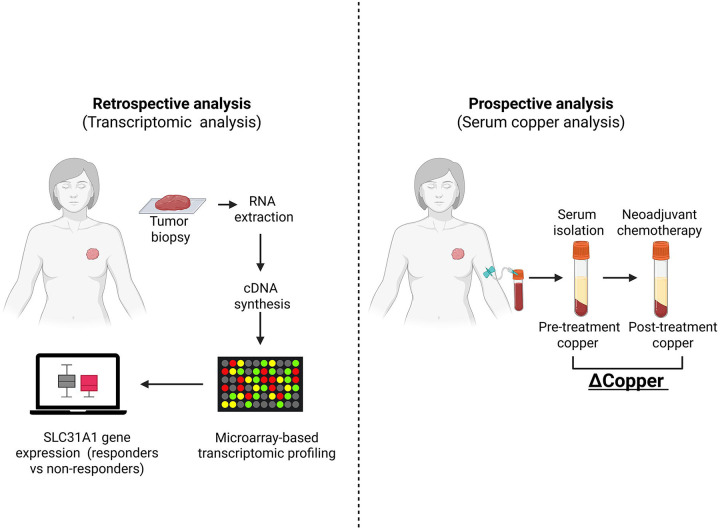
Overview of retrospective and prospective study design. The retrospective arm compared tumor *SLC31A1* expression in responders and non-responders after neoadjuvant therapy, and the prospective arm measured serum copper changes (ΔCopper) before and after neoadjuvant therapy. Abbreviations: Δ, delta.

This study incorporated a retrospective gene-expression analysis and a prospective observational cohort to examine copper biology in breast cancer (Figure 1). The retrospective arm evaluated *SLC31A1* (CTR1) expression in public datasets, while the prospective arm measured serum copper dynamics (ΔCopper) during neoadjuvant therapy. Both datasets were analyzed independently and then compared to identify convergent trends between tissue gene expression and systemic copper changes.

### 2.2 Retrospective analysis of *SLC31A1* gene expression

Expression of *SLC31A1* (CTR1) was analyzed using the ROCplot.org platform,^17^ which integrates transcriptomic and treatment-response data from 36 publicly available breast-cancer datasets. The analysis focused on patients with pathologic response data (n = 1,632) following neoadjuvant chemotherapy. Subgroup analyses were performed by molecular subtype (triple-negative, Luminal A, Luminal B) and by tumor grade within the TNBC cohort. Differences in *SLC31A1* expression between responders and non-responders were assessed using the Mann–Whitney U test, and predictive performance summarized by the area under the receiveroperating-characteristic curve (AUC). In biomarker assessment, AUC values near 0.5 indicate no predictive value; AUC <0.6 reflects limited utility; AUC 0.6–0.7 represents a good biomarker with potential clinical relevance; and AUC >0.7 denotes a high-quality candidate with strong discriminatory performance.

### 2.3 Subtyping and clinical classification

Breast cancer subtypes were defined by estrogen receptor (ER), progesterone receptor (PR), and human epidermal growth factor receptor 2 (HER2) status assessed by immunohistochemistry and confirmed by fluorescence in situ hybridization (FISH). Triple negative breast cancer (TNBC) was defined as ER <1%, PR <1%, and HER2 negative by IHC or FISH. Tumor grade and pathologic stage were from final pathology reports. Treatment response was classified by the treating clinician as responder or non-responder.

### 2.4 Serum collection and copper measurement

Since serum copper is not materially affected by time of day or postprandial interval,^18, 19^ patients were not required to have fasted prior to blood draws, thus minimizing disruption of standard clinical workflow in the neoadjuvant setting.^20^ Peripheral blood was collected by venipuncture at three time points: diagnosis (before therapy), completion of neoadjuvant chemotherapy, and biopsy-confirmed relapse. Serum was separated and stored at −80°C. Total copper was quantified by ICP-MS (Agilent 8900 ICP-QQQ) using standard trace-metal workflows.

### 2.5 Clinical variables captured

Clinical variables were collected as part of routine clinical care and research protocols and are reported in de-identified form.

### 2.6 Primary exposure

ΔCopper (post-pre treatment serum copper, μg/kg) represented the primary exposure. A positive value reflects higher copper after therapy. The first post-therapy sample was used for analysis; later follow-up samples, including the relapse case, were summarized descriptively.

### 2.7 Statistical analysis (prospective cohort)

Data are summarized as medians with interquartile ranges (IQRs); means, minima, and maxima are reported where informative. Associations between ΔCopper and clinicopathologic variables were examined using nonparametric methods. Correlations were evaluated with Spearman’s rank (ρ) and two-sided P values. Group comparisons used Mann–Whitney U tests; comparisons across multiple subtypes used Kruskal–Wallis tests. Analyses were performed in Python (v3.x) with pandas library for data handling, NumPy for numerical computation, and SciPy (scipy.stats) for statistical testing. Figures and graphs were generated in GraphPad Prism (v10).

## 3. Results

### 3.1 Patient characteristics

A total of 21 patients with breast cancer were included in the prospective cohort. Demographic characteristics are summarized in Table 1 and are presented in de-identified form in accordance with preprint data-sharing requirements. Clinical variables were evaluated in relation to serum copper dynamics, tumor subtype, grade, and treatment response and are analyzed in subsequent sections.

### 3.2 Retrospective analysis of *SLC31A1* expression and pathologic response

**Figure 2.**
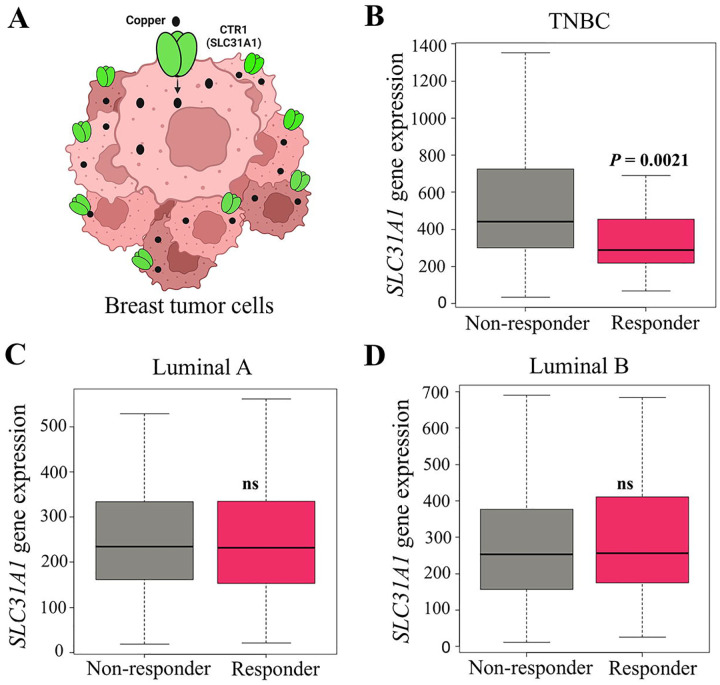
CTR1 function and *SLC31A1* expression by pathologic response. (A) Illustration of CTR1 on the tumor cell surface mediating copper uptake. (B–D) *SLC31A1* transcript levels in responders versus non-responders across breast cancer subtypes. Abbreviations: ns, not significant; TNBC, triple-negative breast cancer.

The integrated study design is shown in Figure 1. To evaluate whether copper import relates to chemotherapy response, *SLC31A1* expression was analyzed in 1,632 breast-cancer patients with pathologic response following neoadjuvant chemotherapy.^17^ Figure 2A illustrates CTR1 localization on tumor cells and its role in facilitating copper uptake.

**Figure 3.**
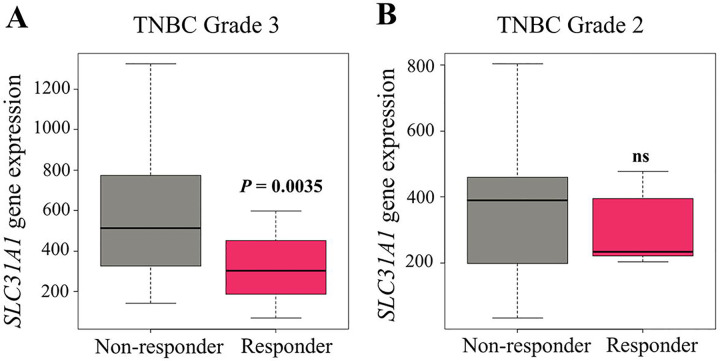
*SLC31A1* expression in TNBC by tumor grade and response. Box plots show *SLC31A1* expression in responders and non-responders with grade 3 (A) or grade 2 (B) TNBC. Abbreviations: ns, not significant; TNBC, triple-negative breast cancer.

Across molecular subtypes, *SLC31A1* expression was highest in triple-negative breast cancer (TNBC) and significantly elevated in non-responders (n = 277) compared with responders (*n* = 196, *P* = 0.0021; Figure 2B; Table 2). The corresponding AUC was 0.682, indicating good predictive potential for distinguishing TNBC non-responders. No significant differences were observed in Luminal A (n = 341 vs 134, *P* = 0.44, AUC = 0.509; Figure 2C) or Luminal B (n = 372 vs 119, *P* = 0.36, AUC = 0.522; Figure 2D; Table 2). Within TNBC, *SLC31A1* expression also varied by histologic grade. In grade 3 tumors, non-responders (*n* = 172) showed significantly higher *SLC31A1* expression than responders (*n* = 89, *P* = 0.0035; Figure 3A; Table 3), with an AUC of 0.717, reflecting stronger discriminatory ability in high-grade disease. In contrast, grade 2 tumors showed no significant difference between non-responders and responders (*n* = 42 vs 13, *P* = 0.54, AUC = 0.576; Figure 3B; Table 3).

These findings indicate that *SLC31A1* upregulation associates with chemotherapy nonresponse and aggressive tumor grade specifically in TNBC and provided the rationale for evaluating dynamic systemic copper changes in the prospective cohort.

### 3.3 Distribution of ΔCopper across breast cancer molecular subtypes

**Figure 4.**
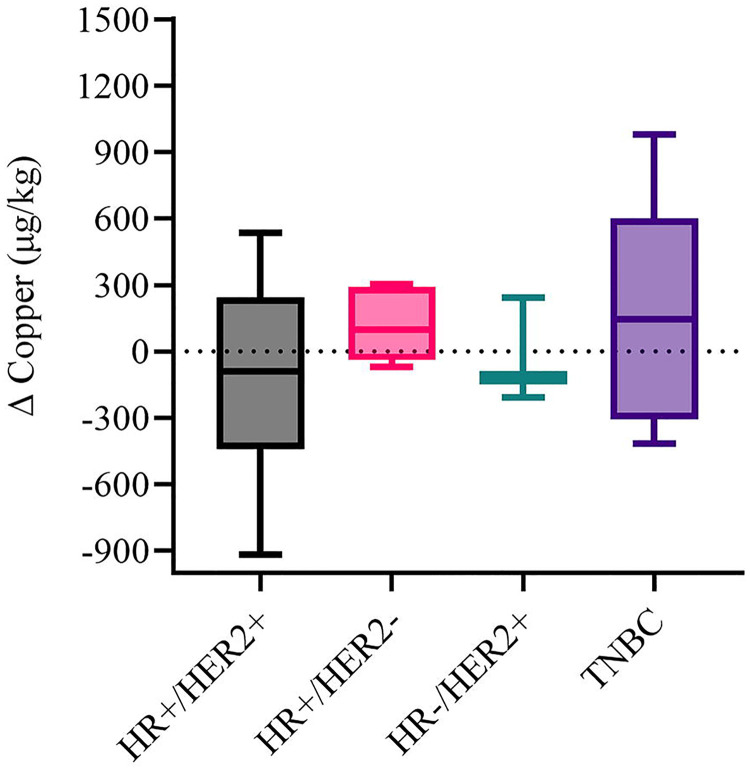
ΔCopper levels in breast cancer patients by molecular subtype. Box plots show the distribution of ΔCopper values (posttreatment − pretreatment) across subtypes. The horizontal line within each box represents the median; box edges represent the interquartile range (IQR); whiskers indicate the minimum and maximum values. Abbreviations: Δ, delta; IQR, interquartile range; TNBC, triple-negative breast cancer.

To explore the dynamic behavior of serum copper following treatment, delta copper (ΔCopper), defined as the change in serum copper concentration from pre- to post-treatment, was quantified across breast cancer molecular subtypes, representing a novel and dynamic metric introduced in this cohort. Median ΔCopper values differed by subtype, with TNBC (n=6) showing the highest median increase of 123.0 μg/kg (interquartile range [IQR], 433.8 μg/kg), followed by HR+/HER2− (n=6) at 98.5 μg/kg (IQR, 263.3 μg/kg), whereas HR+/HER2+ (n=6) and HR−/HER2+ (n=3) exhibited median decreases of −89.5 μg/kg (IQR, 407.5 μg/kg) and −119.0 μg/kg (IQR, 225.5 μg/kg), respectively (Figure 4; Table 4). Although overall distributions did not differ significantly among subtypes (Kruskal–Wallis H = 1.71, *P* = 0.63), TNBC displayed the broadest range of values (−416 to +978 μg/kg) and a trend toward higher ΔCopper compared with other groups.

### 3.4 Distinct ΔCopper pattern in the TNBC relapse case

**Figure 5.**
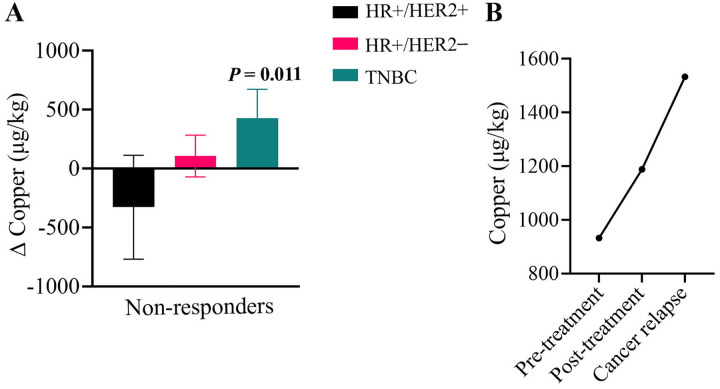
ΔCopper and relapse in breast cancer non-responders. (A) Distribution of ΔCopper values in non-responders across molecular subtypes. (B) Serum copper trajectory of the single TNBC patient who relapsed, showing changes from pretreatment to posttreatment and relapse follow-up. Abbreviations: Δ, delta; TNBC, triple-negative breast cancer.

Because treatment resistance may be associated with distinct copper dynamics, ΔCopper values were examined in non-responders across subtypes (Figure 5). In HR+/HER2+ (n=4) and HR+/HER2− (n=5) subtypes, most non-responders showed near-zero or negative ΔCopper, whereas in TNBC, the single non-responder, who subsequently relapsed, demonstrated consistently positive ΔCopper values at both follow-up timepoints, which were specifically measured to track changes post-treatment and at cancer relapse (Figure 5A). This difference among non- responders reached statistical significance (Spearman ρ = 0.73; *P* = 0.011). In Figure 5B, longitudinal serum copper trajectories for this relapse case showed progressive increases at each timepoint: from 932 μg/kg pre-treatment to 1,188 μg/kg at first follow-up and 1,533 μg/kg at cancer relapse.

### 3.5 Tumor grade correlates with ΔCopper in TNBC

**Figure 6.**
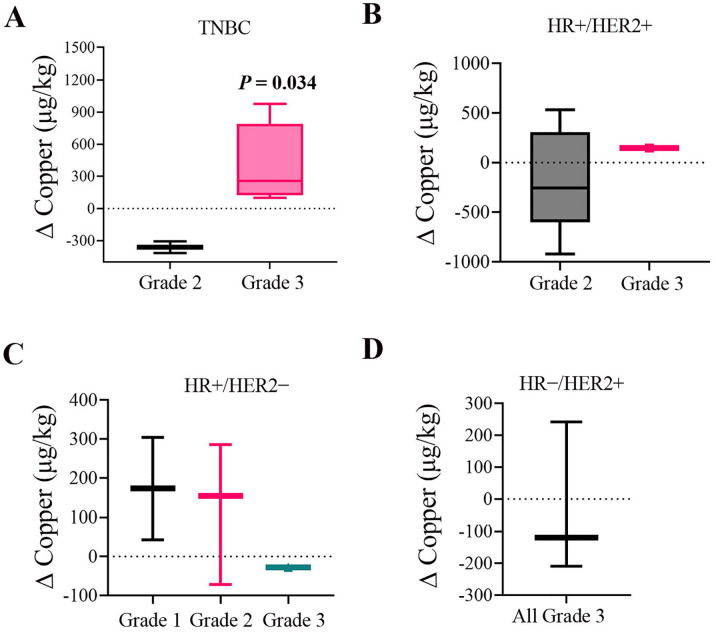
ΔCopper levels stratified by tumor grade within molecular subtypes. Box plots display ΔCopper values (posttreatment − pretreatment) by tumor grade within each molecular subtype: (A) TNBC, (B) HR+/HER2+, (C) HR+/HER2−, and (D) HR−/HER2+. The horizontal line within each box represents the median; box edges indicate the interquartile range (IQR); and whiskers extend to the minimum and maximum values. Abbreviations: Δ, delta; HR, hormone receptor; IQR, interquartile range; TNBC, triple-negative breast cancer.

We next assessed ΔCopper by tumor grade within each subtype, a recognized marker for tumor aggressiveness (Figure 6). In the TNBC group, a striking pattern emerged: all the patients harboring grade 3 tumors (n=4) exhibited positive ΔCopper values, whereas all the patients harboring grade 2 tumors (n=2) displayed negative ΔCopper values (Figure 6A). This association was statistically significant (Spearman ρ = 0.79; *P* = 0.034). Conversely, no consistent or significant correlations were identified between tumor grade and ΔCopper in the HR+/HER2+, HR+/HER2−, or HR−/HER2+ subtypes (Figures 6B–D). These findings highlight a grade-related copper dynamic unique to TNBC in this cohort.

### 3.6 Inverse association between tumor size and pretreatment serum copper

**Figure 7.**
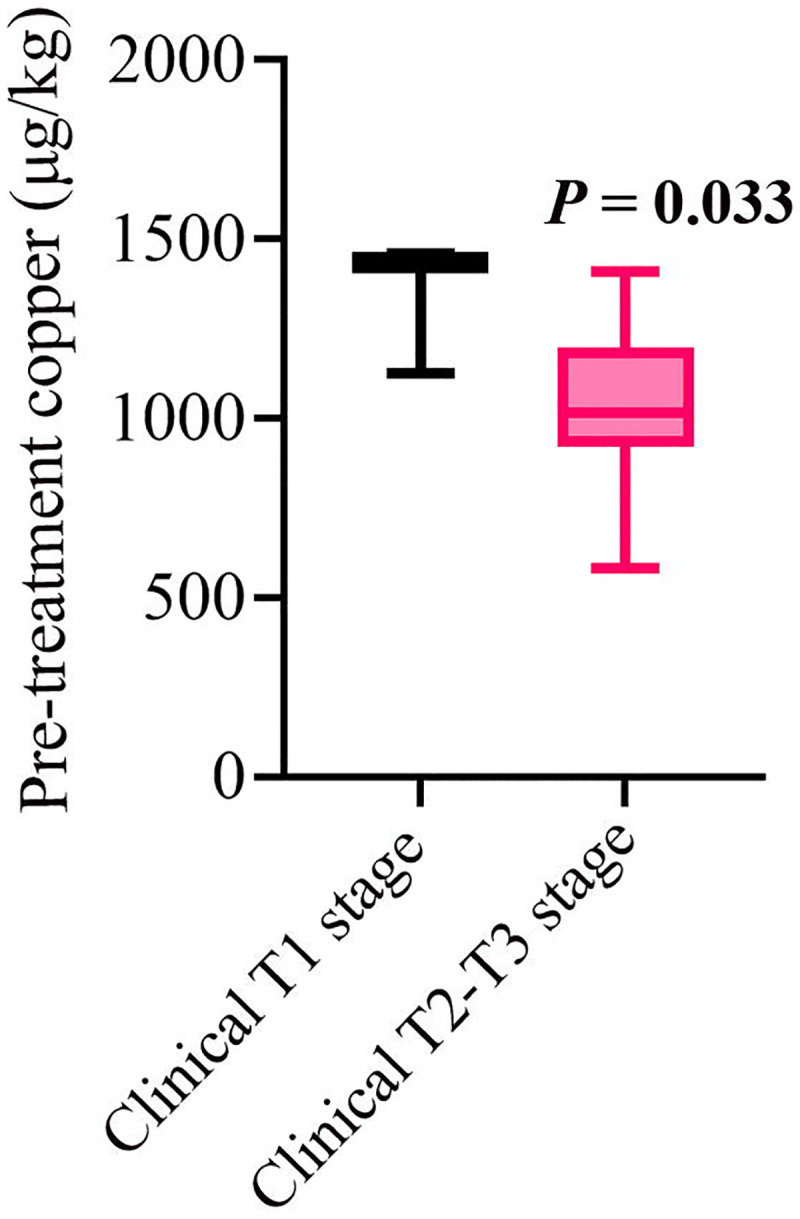
Serum copper levels by clinical T stage. Box plots show serum copper concentrations (μg/kg) for patients with stage T1 (n = 3) and stage T2–T3 (n = 16) breast tumors. The horizontal line within each box indicates the median; box edges represent the interquartile range (IQR); and whiskers denote the minimum and maximum values. Abbreviations: IQR, interquartile range.

We next examined whether pretreatment serum copper levels were associated with clinical tumor size at diagnosis (Figure 7). Prior preclinical studies have reported higher intratumoral copper concentrations in smaller tumors,^10^ and thus we evaluated whether this relationship extended to systemic serum copper. Female patients were grouped by clinical tumor stage as having T1 tumors (≤2 cm; n = 3) or T2–T3 tumors (>2 cm; n = 16). Median pretreatment serum copper levels were significantly higher in patients with smaller T1 tumors compared with those harboring larger T2–T3 tumors (1,435 μg/kg [IQR, 166 μg/kg] vs 1,015 μg/kg [IQR, 268 μg/kg]; Mann-Whitney U = 43, *P* = 0.033).

## 4. Discussion

Copper supports multiple metabolic and signaling processes essential to cancer progression, including mitochondrial respiration, redox maintenance, angiogenesis, and extracellular matrix (ECM) remodeling.^1^ Clinical studies of systemic copper chelation in high-risk triple-negative breast cancer (TNBC) suggest that copper metabolism can be therapeutically targeted in this subtype.^16^ However, key questions remain: Why does TNBC show such a strong association with copper biology? Is copper linked to tumor aggressiveness and resistance to neoadjuvant chemotherapy? And can copper-associated biomarkers guide personalized treatment or copper-chelation strategies?

To begin addressing these questions, we conducted the first integrated analysis combining paired serum copper measurements in a prospective neoadjuvant setting with retrospective tumor CTR1 copper importer gene expression linked to neoadjuvant chemotherapy response. This allowed us to assess copper uptake at the tumor level (CTR1) alongside dynamic systemic copper changes (ΔCopper) during treatment, an aspect of copper biology not previously evaluated in breast cancer patients.

A consistent and clinically meaningful pattern emerged across both datasets: high-grade TNBC engages a coordinated copper axis. In the retrospective analysis of 1,632 patients, CTR1 (*SLC31A1*) expression was significantly elevated only in grade 3 TNBC non-responders, with no differences in grade 2 TNBC or luminal subtypes. In the prospective cohort, ΔCopper behavior mirrored this specificity: all grade 3 TNBCs demonstrated positive ΔCopper, whereas all grade 2 TNBCs showed negative ΔCopper, and non-responders from other subtypes tended to maintain or reduce systemic copper levels. The only TNBC non-responder, who later relapsed, displayed persistently rising ΔCopper across the sampling timeline. Taken together, these findings suggest that copper metabolism is tightly linked to both tumor aggressiveness and treatment resistance in high-grade TNBC.

Several biologically plausible mechanisms may explain this pattern. Copper is required for enzymes and signaling mediators such as cytochrome c oxidase, superoxide dismutase, the lysyl oxidase (LOX/LOXL1–4) family, and kinases including MEK1 and ULK1/2. These copper-dependent activities support several processes fundamental to cancer cell survival and progression.^1^ Increased copper import through CTR1 could enhance the activity of these or other copper-dependent pathways, contributing to metabolic fitness, redox stability, autophagy regulation, and ECM remodeling, processes that may collectively promote aggressive tumor behavior and could attenuate chemotherapy efficacy. The LOX family is particularly relevant: LOX-mediated collagen crosslinking has been implicated in chemotherapy resistance in TNBC, and LOX inhibition can re-sensitize resistant models.^21^ Increased copper availability may enhance LOX activity and ECM-mediated survival pathways, providing a plausible mechanism linking copper metabolism to treatment resistance in high-grade TNBC.

We also observed that patients with smaller primary tumors had higher pretreatment serum copper, aligning with the hypothesis that biologically active, early-stage tumors may trigger copper mobilization from copper-rich sources such as the liver to meet angiogenic and metabolic demands. This pattern parallels preclinical findings of elevated intratumoral copper in smaller tumors.^10^ If validated, pretreatment copper levels and ΔCopper trajectories could serve as stage- or biology-informed biomarkers.

From a translational perspective, combining tumor CTR1 expression with ΔCopper dynamics emerges as a promising biomarker profile. This integrated copper signature could help identify patients with copper-dependent and treatment-resistant biology, particularly within high-grade TNBC and may guide the selection of patients for copper-modulating therapeutic strategies. These findings also support the concept that copper biomarkers may complement existing clinicopathologic tools for risk stratification in the neoadjuvant setting.

Although the prospective cohort size is modest, the reproducible, subtype- and grade-specific patterns observed across two independent datasets provide a strong basis for further investigation. Future studies should expand these observations in larger cohorts and incorporate ΔCopper alongside tumor profiling of CTR1, copper-dependent enzymes, and copper-regulated signaling pathways to refine patient selection and clarify mechanisms linking copper metabolism to neoadjuvant chemotherapy resistance.

In summary, this integrated analysis provides the first clinical evidence that high-grade TNBC activates a coordinated tumor-systemic copper program characterized by elevated CTR1 expression and increased ΔCopper during neoadjuvant therapy. These hypothesis-generating findings strengthen the emerging link between copper metabolism, tumor aggressiveness, and treatment resistance, and establish a foundation for incorporating copper-related biomarkers into future personalized therapeutic strategies and clinical trial designs.

## Figures and Tables

**Table 1.** Demographic characteristics of breast cancer patients (N = 21)

Patient ID	Age range (years)	Sex
BCP-001	60–64	F
BCP-002	60–64	F
BCP-003	35–39	F
BCP-004	25–29	F
BCP-005	60–64	F
BCP-006	35–39	F
BCP-007	50–54	F
BCP-008	50–54	F
BCP-009	35–39	F
BCP-010	70–74	F
BCP-011	40–44	F
BCP-012	60–64	F
BCP-013	35–39	F
BCP-014	55–59	F
BCP-015	70–74	F
BCP-016	70–74	F
BCP-017	55–59	F
BCP-018	60–64	F
BCP-019	50–54	F
BCP-020	45–49	F
BCP-021	70–74	M

Ages are presented as non-overlapping 5 year ranges to minimize the risk of participant reidentification. Patient IDs shown are anonymized for presentation and do not correspond to original identifiers.

**Table 2.** Sample distribution and statistical comparison for *SLC31A1* analysis across breast cancer subtypes

Subtype	Non-responders (n)	Responders (n)	P value (Mann Whitney U)
TNBC	277	196	0.0021
Luminal A	341	134	Not significant
Luminal B	372	119	Not significant

Abbreviations: TNBC, triple-negative breast cancer.

**Table 3.** Sample distribution and statistical comparison for *SLC31A1* analysis by tumor grade in triple-negative breast cancer.

Subtype/Grade	Non-responders (n)	Responders (n)	P value (Mann Whitney U)
TNBC grade 3	172	89	0.0035
TNBC grade 2	42	13	0.54 (not significant)

Abbreviations: TNBC, triple-negative breast cancer.

**Table 4.** ΔCopper levels in breast cancer patients by molecular subtype (N=21)

Subtype	n	Median ΔCu μg/kg	Mean ΔCu μg/kg	IQR	Min	Max
HR+/HER2+	6	−89.5	−116.8	407.5	−919	534
HR+/HER2−	6	98.5	114.5	263.3	−72	304
HR−/HER2+	3	−119.0	−28.7	225.5	−209	242
TNBC	6	123.0	126.2	433.8	−416	978
Total	21					

Abbreviations: Cu, copper; HER2, human epidermal growth factor receptor 2; HR, hormone receptor; IQR, interquartile range; TNBC, triple-negative breast cancer.

## Data Availability

The prospective clinical data supporting the findings of this study are not publicly available due to patient privacy and ethical considerations but may be made available from the corresponding author upon reasonable request and with appropriate institutional approvals. Publicly available gene expression datasets analyzed in this study are accessible through ROCplot.org.
